# Emergence and genomic characterization of hypervirulent ST23/K1 *Klebsiella pneumoniae*: local epidemiology and global context

**DOI:** 10.3389/fmicb.2026.1758288

**Published:** 2026-02-02

**Authors:** Matej Bezdicek, Marketa Jakubickova, Viktoria Bitusikova, Ema Holubova, Helena Vitkova, Iva Kocmanova, Ivana Vitkova, Lenka Zdrazilova Dubska, Martina Lengerova

**Affiliations:** 1 Division of Clinical Microbiology and Immunology, Department of Laboratory Medicine, University Hospital Brno, Brno, Czechia; 2 Division of Clinical Microbiology and Immunology, Department of Laboratory Medicine, Faculty of Medicine, Masaryk University, Brno, Czechia; 3 Department of Biomedical Engineering, Faculty of Electrical Engineering and Communication, Brno University of Technology, Brno, Czechia

**Keywords:** hypervirulent, *Klebsiella pneumoniae*, long-read sequencing, multidrug resistance, plasmid, ST23, whole genome sequencing

## Abstract

**Introduction:**

Hypervirulent *Klebsiella pneumoniae* (hvKp) of the K1/ST23 lineage is an emerging global threat associated with invasive community-acquired infections. Increasing reports of virulence–resistance convergence highlight the need for genomic surveillance, particularly within Europe where data remain limited. This study characterizes clinical K1/ST23 KP isolates circulating in the Czech Republic and compares them to a global genomic background to evaluate virulence architecture, resistance acquisition and plasmid evolution.

**Methods:**

From 2017 to 2025, 570 *K. pneumoniae* isolates from a tertiary-care hospital were screened for hvKp markers. Ninety-six K1/ST23 isolates were subjected to long-read whole-genome sequencing and plasmid reconstruction. Genomes were analyzed alongside 2,463 international ST23 datasets using core-SNV phylogenomics, virulence/resistance profiling, and structural plasmid mapping. Chromosomal integrations were examined through analysis of flanking insertion-sequence contexts.

**Results:**

The Czech K1/ST23 KP population exhibited high virulence uniformity (95/96 isolates scoring 9/9) without evidence of a single-clone outbreak, instead forming multiple phylogenetic lineages consistent with recurrent introductions. Eighty-three isolates carried pLVPK-like virulence plasmids; however, structural plasticity was prominent. The *iro* cluster was relocated to conjugative IncFII/rep_cluster_1418 plasmids in two isolates—one carrying additional AMR genes—and was chromosomally integrated via IS1-mediated recombination in three others. Iut was chromosomally integrated via IS903 (IS5 family) with either classical target-site duplication or recombination-associated insertion. Nine virulence–resistance fusion plasmids (IncFIB–IncFII–IncHI1B or IncC-based) were identified, representing early convergence toward MDR-hvKp.

**Conclusion:**

K1/ST23 KP circulating in the Czech Republic is highly virulent yet genomically diverse, driven by active plasmid exchange, insertion-sequence–mediated chromosomal integration, and emerging virulence–resistance fusion plasmids. Although carbapenemase genes were absent, ESBL determinants and transmissible virulence loci indicate strong evolutionary potential toward MDR-hvKp. Continuous genomic surveillance and early intervention strategies are essential to mitigate future clinical impact.

## 1 Introduction

Currently, two evolutionary lineages of *Klebsiella pneumoniae* are recognized, each associated with distinct clinical and epidemiological features (Russo and Marr, 2019; García-Cobos et al., 2025). The first is referred to as classical *K. pneumoniae* (cKp), a typical opportunistic pathogen responsible for infections commonly associated with healthcare settings and often linked to extensive antimicrobial resistance. The second lineage comprises hypervirulent *K. pneumoniae* (hvKp) strains, which often cause community-acquired infections in otherwise healthy individuals of any age (Russo and Marr, 2019; Mendes et al., 2023). A typical hvKp clinical manifestation is a pyogenic liver abscess with no underlying biliary disease, frequently accompanied by metastatic spread (Russo and Marr, 2019; Shankar et al., 2020; Mendes et al., 2023). Additional manifestations, such as meningitis, brain abscess, and pneumonia may occur in some patients (Paczosa et al., 2020; Shankar et al., 2020).

Although historically concentrated in the Asia-Pacific region, hvKp has now been reported worldwide (Zhu et al., 2021). Simultaneously, its definition has evolved; initially associated with a hypermucoviscous phenotype, it is now understood that not all hvKp strains are hypermucoviscous, and some cKp strains can exhibit this trait, making hypermucoviscosity alone insufficient for definitive identification (Russo et al., 2024a). Instead, hvKp is currently defined by the presence of a specific set of virulence genes. Key determinants include increased capsule production (*rmpA*/*rmpA2*), aerobactin (*iuc*), salmochelin (*iro*), yersiniabactin (*ybt*), and colibactin (*clb*) (Chen et al., 2023; Russo et al., 2024a; García-Cobos et al., 2025). Among these, aerobactin and genes such as *iucA*, *iroB*, and *peg-344* are considered more reliable biomarkers than hypermucoviscosity alone (Russo et al., 2024a,b).

A major concern regarding hvKp is the convergence of hypervirulence and antimicrobial resistance, particularly carbapenem resistance, resulting in multidrug-resistant (MDR) or carbapenem-resistant hvKp (CR-hvKP) (Tang et al., 2020; Biedrzycka et al., 2022; Gálvez-Silva et al., 2024). This convergence typically occurs either through hvKp acquiring resistance plasmids or through transferring virulence plasmids into classical MDR lineages (Russo and Marr, 2019; Mendes et al., 2023). Such hybrid strains combine high pathogenicity with limited treatment options, posing a significant clinical threat.

Despite being recognized as a global threat by the WHO (World Health Organization, 2024), substantial knowledge gaps remain regarding hvKp’s global prevalence, community acquisition mechanisms, and optimal infection control strategies. The hvKp infections, particularly those caused by MDR-hvKp, require early recognition, source control, and site-specific antimicrobial regimens. Routine clinical microbiology laboratories often lack tools to reliably differentiate hvKp from cKp, which complicates early recognition and management. Expanded genomic surveillance integrating virulence and resistance profiling is therefore essential to improve detection and track dissemination of this high-risk pathogen (Russo et al., 2024a).

In this study, we specifically focused on the globally dominant hvKp clone ST23 carrying capsular type K1 (K1/ST23 KP), which represents the most successful and clinically relevant hvKp lineage worldwide. We characterized a collection of K1/ST23 KP isolates obtained between 2017 and 2025 from the Czech Republic, aiming to define their genomic diversity, virulence landscape, and antimicrobial susceptibility profiles. An essential objective in our investigation was to determine whether these isolates represented a localized long-term outbreak or independent introductions, as this distinction is critical to interpret current findings and guide future K1/ST23 KP surveillance within the region. Utilizing long-read sequencing, we compared these isolates with a global K1/ST23 KP dataset to contextualize local epidemiology within the broader global population structure. Together, these findings contribute to a more comprehensive understanding of K1/ST23 KP dynamics in Central Europe and form future public health strategies focused on early detection and prevention.

## 2 Materials and methods

### 2.1 Bacterial isolate collection

A total of 570 *Klebsiella pneumoniae* isolates collected at University Hospital Brno (UHB), Czech Republic, between April 2017 and August 2025 were prospectively screened for suspected hypervirulence (defined as the presence of a hypermucoviscous phenotype, an invasive clinical presentation, or both). All isolates were identified using MALDI-TOF mass spectrometry and typed using a combination of qualitative multiplex PCR targeting virulence genes (Compain et al., 2014) and a rapid typing method mini-MLST (Andersson et al., 2012), which allows isolates to cluster into groups correlating with sequence types without the need for sequencing. All isolates were stored at −80 °C in glycerol stocks.

### 2.2 Genome sequencing, assembly and annotation

High-molecular-weight genomic DNA for long-read sequencing was extracted using the DNeasy PowerSoil Pro Kit (Qiagen, NL) following the manufacturer’s instructions. Although not primarily optimized for ultra–high-molecular-weight DNA, this approach consistently yielded sufficient read lengths and coverage for complete genome and plasmid assembly in *K. pneumoniae*. DNA purity was assessed with a NanoDrop spectrophotometer (Thermo Fisher Scientific, United States), and concentration was measured using a Qubit 4.0 Fluorometer (Thermo Fisher Scientific, United States). Library preparation was performed using the Rapid Barcoding Kit 96 V14 (Oxford Nanopore Technologies, UK), and sequencing was carried out on R10.4.1 flow cell using the PromethION 2 Solo platform (Oxford Nanopore Technologies, UK).

Sequencing data were basecalled with Dorado integrated in MinKNOW (v25.05.14) using the super-accurate model (dna_r10.4.1_e8.2_400bps_sup@v5.0.0). Reads with a quality score ≥10 were retained for downstream processing. The Q ≥ 10 threshold was selected based on internal benchmarking, which showed no relevant impact on SNV detection while improving assembly completeness compared to more stringent cutoffs. Demultiplexing and adapter trimming were repeated using Dorado. As part of our standardized bioinformatic pipeline, reads were mapped to the human reference genome (GCF_000001405.40) using minimap2 (v2.28) (Li, 2018) to remove any non-bacterial sequences, including residual host DNA and potential contaminating DNA introduced during sample handling and library preparation. Next, unmapped reads were extracted with Samtools (v1.19) (Danecek et al., 2021). *De novo* genome assembly was performed using Flye (v2.9.3) (Kolmogorov et al., 2019). Assemblies were polished using the two internal Flye polishing iterations; no additional polishing steps were applied based on internal comparisons against hybrid reference assemblies.

### 2.3 External genomes collection

In addition to the local Czech isolates, we analyzed 2,463 publicly available K1/ST23 KP genomes retrieved from NCBI^1^ and the Institut Pasteur BIGSdb database.^2^ Using Kleborate (v3.1.3) (Lam et al., 2021), we confirmed that all genomes belonged to the K1/ST23 lineage, enabling direct comparison with the UHB isolates. A complete list of genomes included in this study, together with basic metadata (country and year of isolation, sample type, and assembly metrics), is provided in Supplementary Table S1.

### 2.4 Phylogenomic analysis

Core genome SNVs (cgSNVs) analysis of the 96 UHB isolates was performed in Ridom SeqSphere+ (Ridom, DE). A total of 1,229 high-quality cgSNV positions were extracted using the integrated *K. pneumoniae* cgMLST scheme, which comprises 2,358 target genes; the maximum pairwise distance observed was 144 SNVs, spanning a total alignment length of 1,955,621 bp. The resulting UPGMA phylogenetic tree was used for exploratory local clustering of closely related isolates and was exported and visualized in iTOL (v7.2.2) (Letunic and Bork, 2024).

For the extended dataset’s phylogenomic comparison (*n* = 2,559 K1/ST23 KP isolates), whole-genome alignment against the K1/ST23 KP reference genome SGH10 (Lam et al., 2018) was performed by MUMmer (v4.0.1) (Marçais et al., 2018). Genomes were annotated with DFAST (v1.3.5) (Tanizawa et al., 2018), and core genes were identified with Roary (v3.13.0) (Page et al., 2015). A custom Python script was used to extract a predefined set of core genes (659 core genes present in all (100%) analyzed strains), retrieve their genomic coordinates, and integrate them with SNVs identified by MUMmer; these core genes spanned a total alignment length of 472,437 bp. Based on 8,761 core cgSNVs, a maximum-likelihood phylogenetic tree was constructed using IQ-TREE (v3.0.1) (Wong et al., 2025) with 1,000 bootstrap replicates. Model selection was performed using ModelFinder, and the best-fit substitution model was the transversion model with empirical base frequencies (TVM + F). The final phylogeny was visualized in iTOL.

### 2.5 Antimicrobial resistance and virulence analysis

AMR genes were identified using ResFinder (v4.6.0) (Bortolaia et al., 2020). Virulence genes were detected using VirulenceFinder (v3.0.1) (Joensen et al., 2014) and Kleborate (v3.1.3) (Lam et al., 2021). Specific virulence markers, including *allS*, *mceG*, and *peg-344* were additionally searched by BLAST (v2.12) (Altschul, 1997).

### 2.6 Plasmid analysis

Plasmid identification and classification were performed using a stepwise workflow integrating contig topology, replication-associated genes, sequence similarity, coverage patterns, and functional annotation (Supplementary Figure S1). Following *de novo* assembly, all contigs were retained and initially categorized based on circularity and size. Circular plasmids were directly subjected to downstream plasmid analyses. Linear contigs were screened for replication markers using the MOB-suite (v3.1.9) (Robertson and Nash, 2018) and evaluated using NCBI BLAST and reference-based mapping in Proksee (Grant et al., 2023). Reference plasmids included plasmids from other isolates as well as plasmids identified in preceding BLAST-based steps. During reference-based mapping, contig coverage was taken into account, and contigs originating from the same isolate and mapping to a single reference were evaluated collectively.

MOB-suite was applied iteratively to all 194 plasmid-associated contigs obtained from 96 UHB isolates, including complete circular plasmids, linear contigs with identified replication types, and concatenated plasmid sequences reconstructed through reference-based mapping. Short linear contigs (<5 kbp) not assigned to either chromosome or plasmid at any step were excluded from subsequent analyses. Final plasmid classification and annotation were performed using MOB-suite, Bakta, and CARD.

## 3 Results

**Figure 1:**
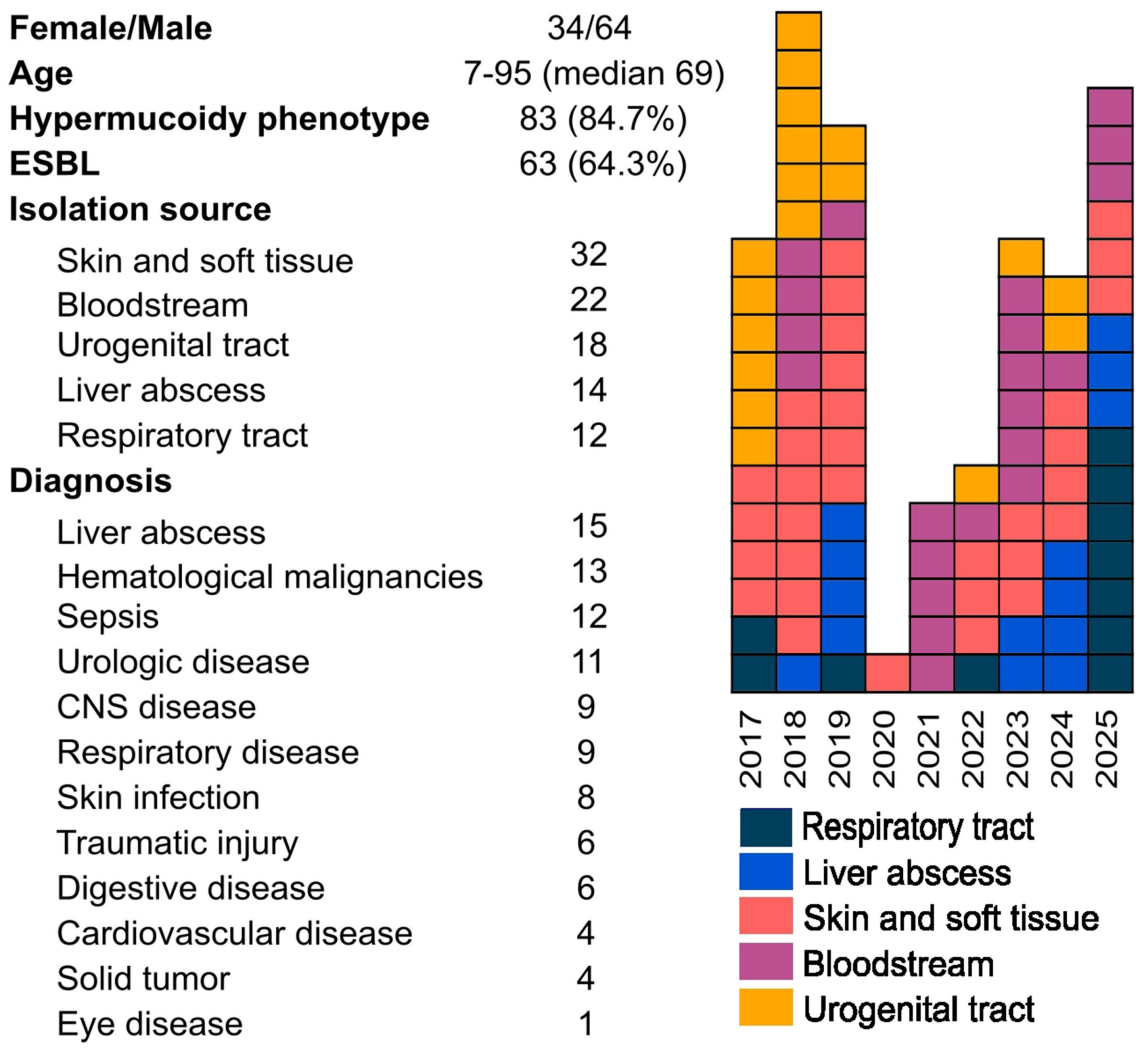
Clinical metadata and temporal distribution of K1/ST23 isolates collected at University Hospital Brno between 2017 and 2025. Annual isolate counts show fluctuating detection frequency during the study period, including a marked decline in 2020–2022 coinciding with the COVID-19 pandemic.

Out of 570 *K. pneumoniae* isolates screened, 96 isolates originating from 82 patients met the combined molecular and genotypic criteria defining the K1/ST23 lineage. These isolates exhibited a conserved multiplex PCR virulence profile (*magA*, *iutA*, *allS*, *kfu*, *rmpA*, *entB*, *mrkD*, and *ybtS*) and melt-type 281, which corresponds to sequence type ST23. WGS-based *in silico* MLST confirmed ST23 assignment for all isolates with full concordance. Among these 96 isolates, 62 (63.5%) exhibited an ESBL phenotype, 81 (84.4%) exhibited a hypermucoid phenotype confirmed by a positive string test, and none showed carbapenem resistance. A comprehensive overview of epidemiological characteristics is shown in Figure 1. Regarding the available metadata for the global dataset of 2,463 isolates, these are provided in the Supplementary Table S1 and include the country and city of origin, year of isolation, and sample type, along with basic assembly information.

### 3.1 Phylogenetic analysis

**Figure 2:**
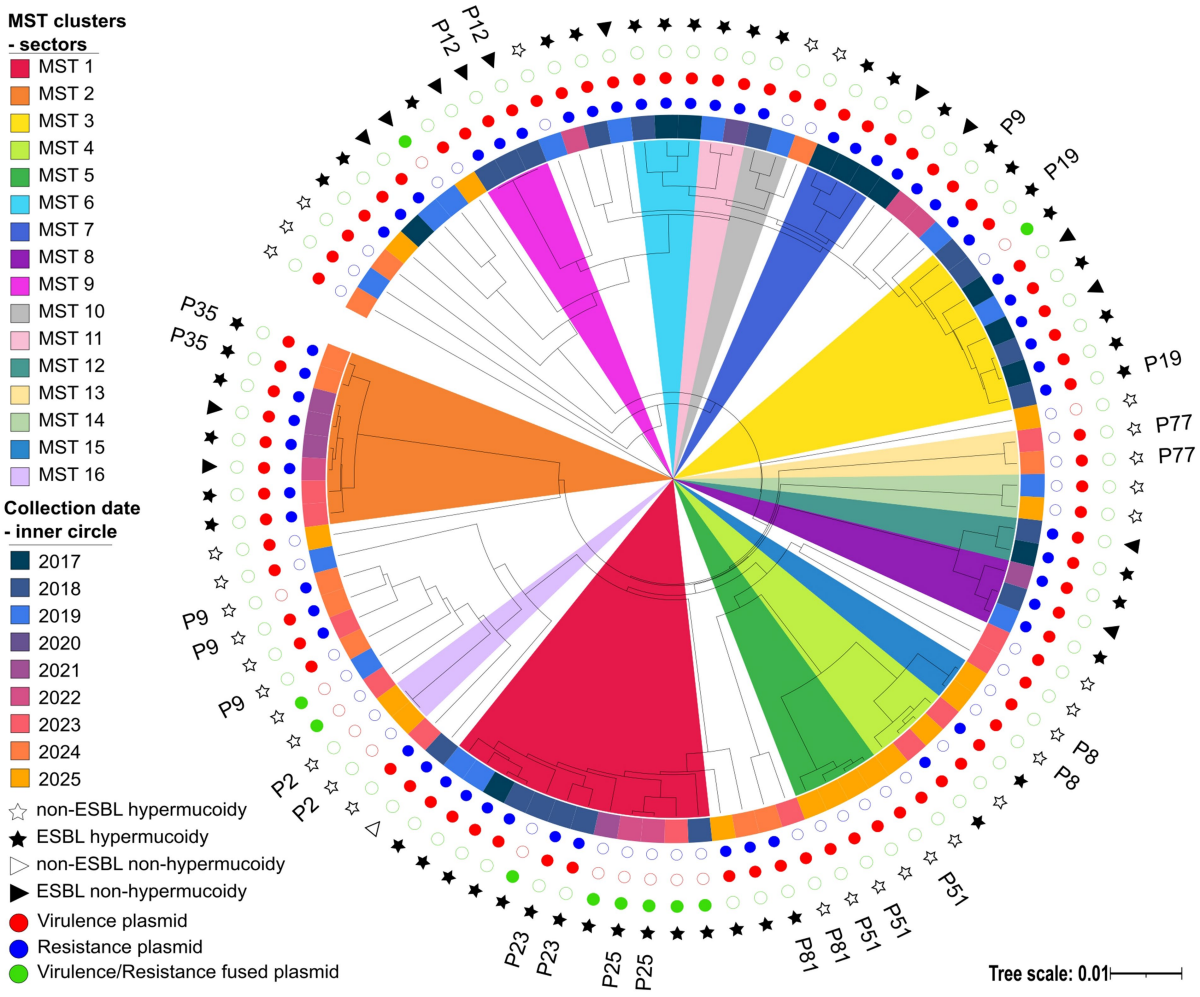
Maximum-likelihood core-genome SNV phylogeny of Czech K1/ST23 isolates based on 1,229 core-genome SNVs. Plasmid carriage is indicated by coloured dots: blue for resistance plasmids, red for virulence plasmids, and green for fusion plasmids. The inner ring denotes collection year, with coloured sectors distinguishing MST clusters; the outer ring represents phenotypic characteristics.

Core-genome phylogenetic reconstruction based on 1,229 SNVs demonstrated substantial genomic diversity within the K1/ST23 KP population. Using a ≤ 10-SNV threshold, we identified 16 phylogenetic clusters (2 to 12 isolates each) alongside 34 unique singletons. Isolates from the same patient consistently clustered together, with one exception in patient 9, where an isolate from 2022 diverged markedly from three isolates obtained in 2024, suggesting either within-host diversification, long-term colonization dynamics, or reinfection by a genetically distinct ST23 variant. Figure 2 depicts phylogenetic relationships among UHB isolates, annotated with phenotypes, plasmid composition, and isolation dates. Major clusters are highlighted, providing a summary of phenotypic traits, plasmid content, and sampling date. For a detailed visualization of pairwise SNV distances among isolates, including the corresponding minimum-spanning tree, please refer to the Supplementary Figure S2.

**Figure 3:**
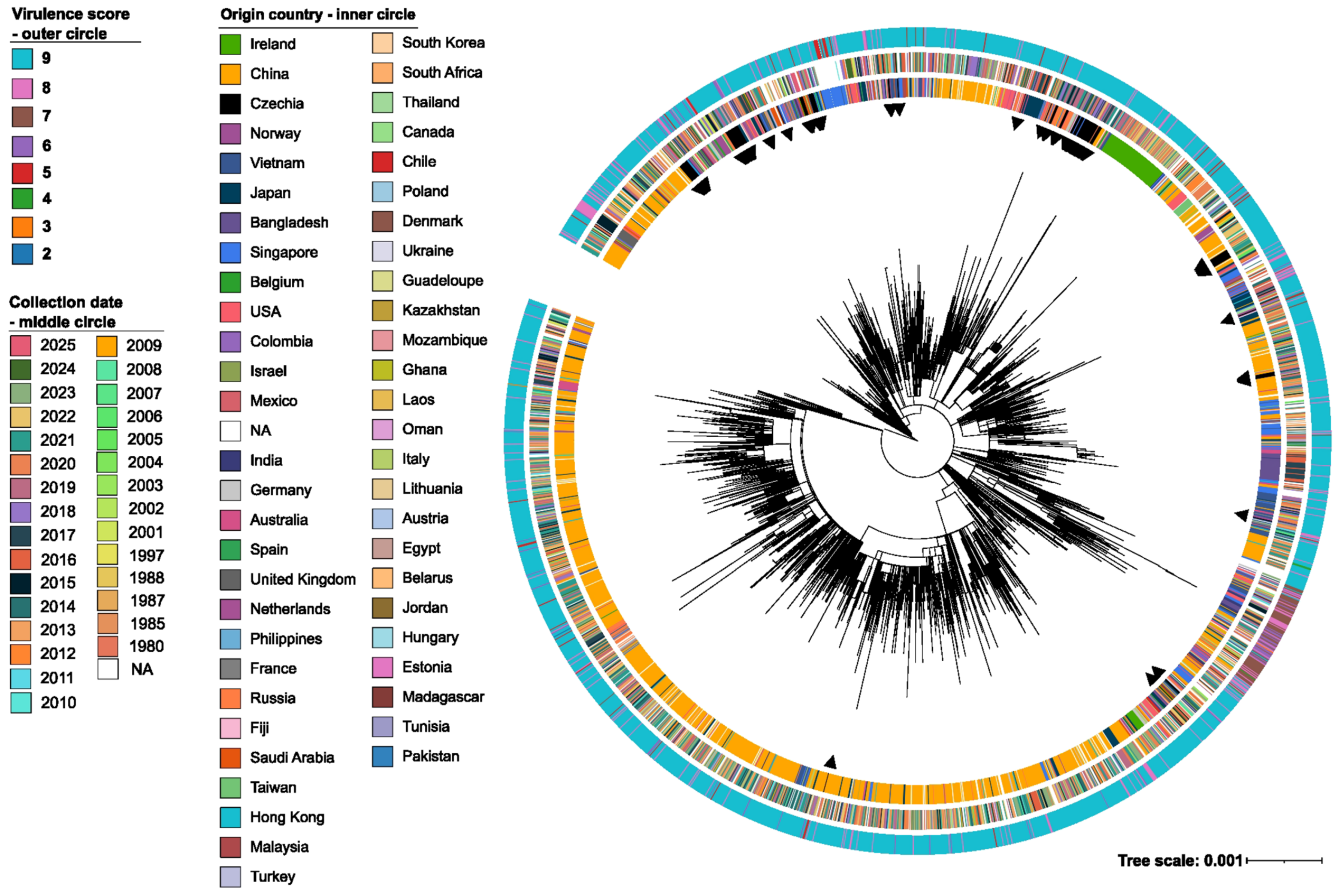
Maximum-likelihood core-genome SNV phylogeny of 2,463 global K1/ST23 genomes and 96 Czech K1/ST23 isolates, based on 8,761 core-genome SNVs. Black triangles indicate Czech isolates. The inner ring represents country of origin, the middle ring indicates collection year, and the outer ring displays virulence scores.

A second phylogenetic analysis including 96 UHB isolates and 2,463 publicly available K1/ST23 KP genomes based on 8,761 core-genome SNVs revealed that UHB isolates were scattered across multiple branches rather than forming a single monophyletic cluster (Figure 3). This distribution indicates repeated K1/ST23 KP introductions into the Czech population rather than single lineage clonal expansion.

**Figure 5:**
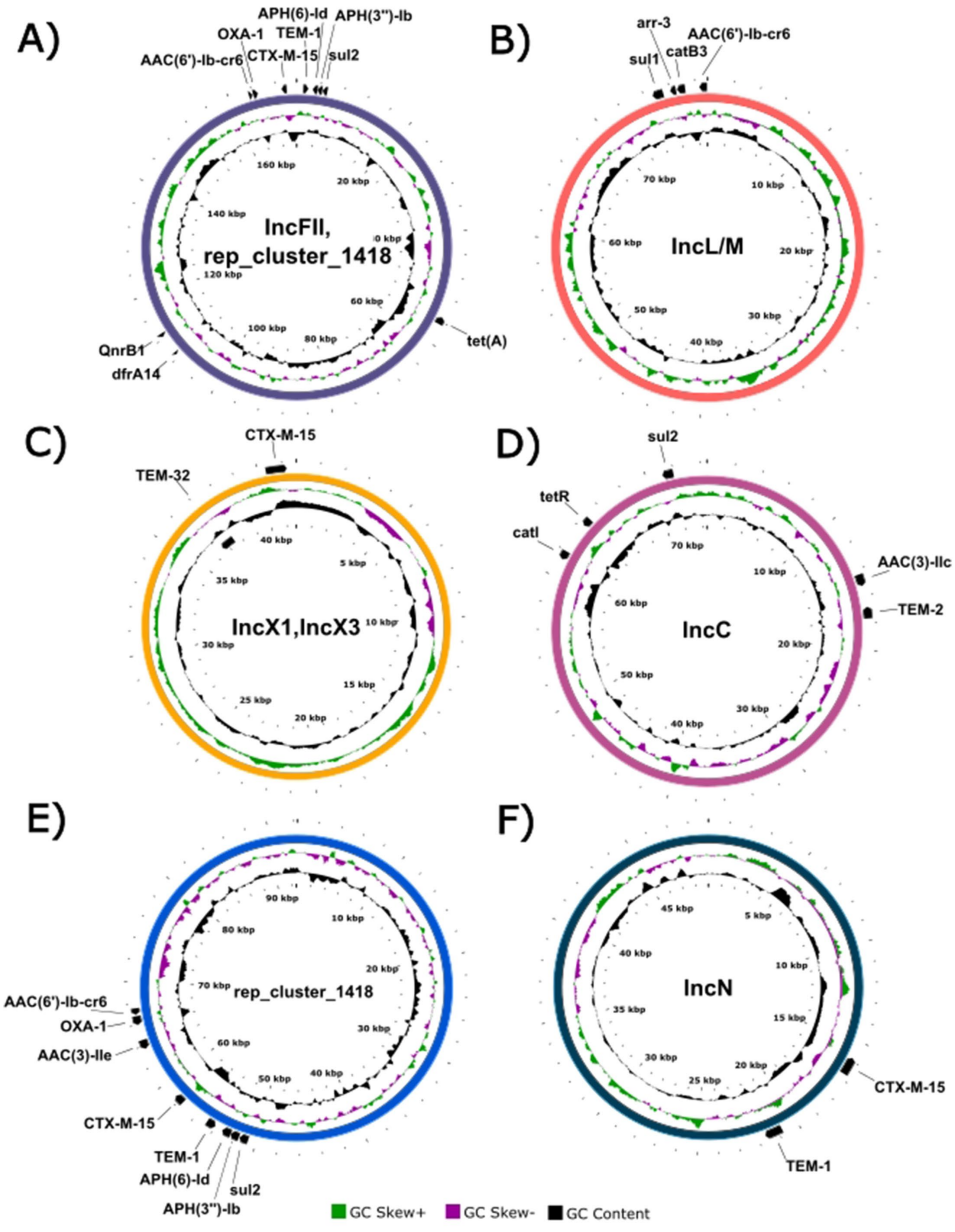
Proksee visualization of resistance plasmid replicon types across Czech K1/ST23 isolates. Annotated resistance genes are shown.

### 3.2 Plasmid analysis

**Figure 4:**
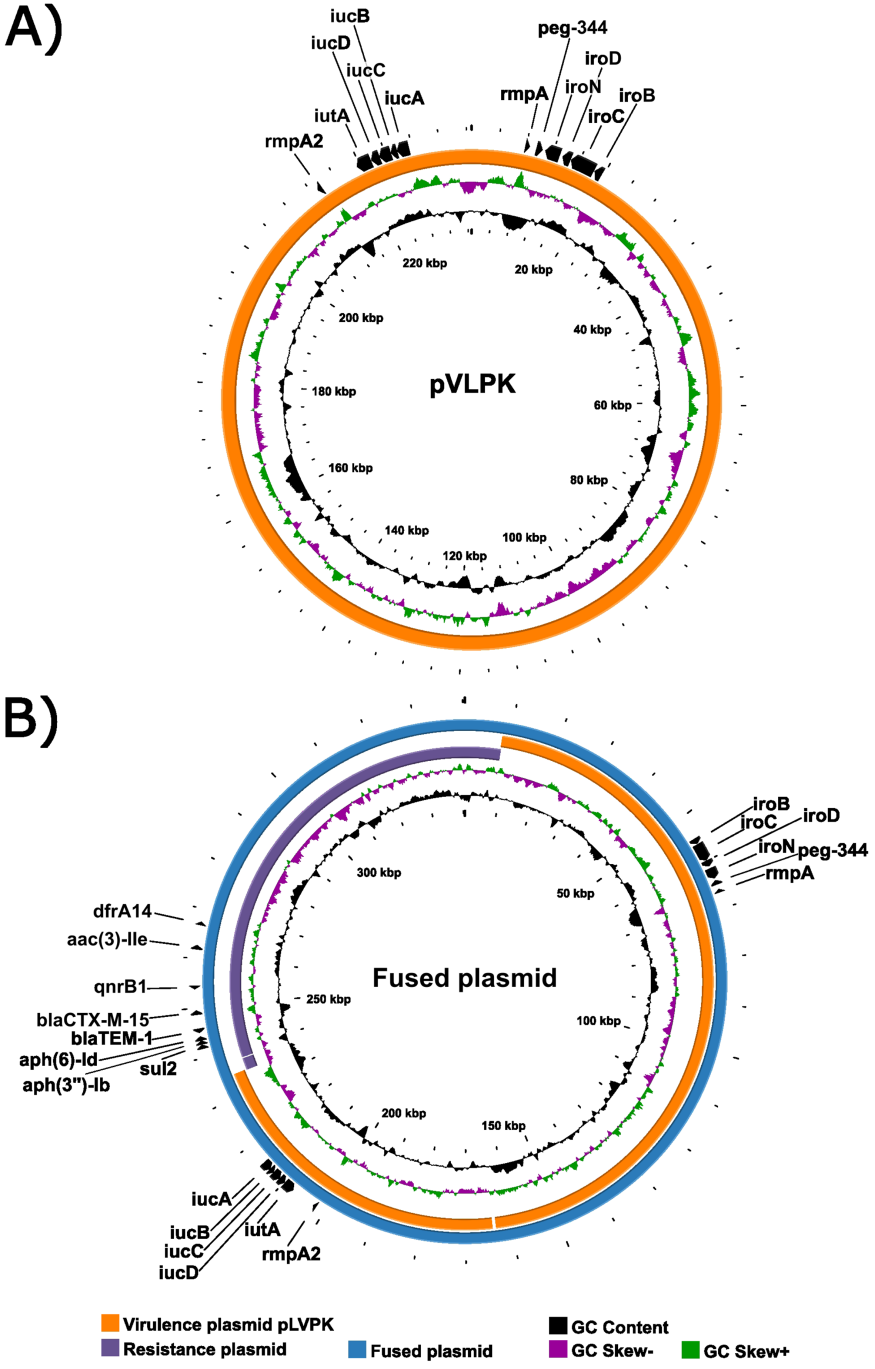
Proksee maps of pLVPK-like virulence plasmids **(A)** and virulence–resistance fusion plasmids **(B)**. Annotated virulence and resistance genes are indicated. In panel **B**, the IncFII/rep_cluster_1418 resistance plasmid and pLVPK reference sequence are mapped to the fusion plasmid backbone.

Plasmid reconstruction revealed a complex and heterogeneous plasmidome K1/ST23 lineage characteristic (Figures 4, 5). The median number of plasmids per isolate was 2, with a total range of 0–5, reflecting substantial diversity in plasmid carriage across the population (for more detailed information see Supplementary Tables S2, S3).

Virulence plasmids were widespread, with 92/96 (95.8%) isolates carrying pLVPK-like plasmids belonging to the IncFIB/IncHI1B type. These plasmids harbored the canonical virulence loci *iro*, *iut*, *rmpA*, *rmpA2*, and *peg-344* and displayed a highly conserved backbone structure (Figure 4). For complete alignment of all pLVPK-like plasmids detected in our collection (see Supplementary Figure S3). Importantly, the *iro* gene cluster in 5/92 isolates was not located on the canonical pLVPK plasmid. Instead, in two of these five isolates the *iro* cluster, *rmpA* and *peg-344* resided on an IncFII/rep_cluster_1418 plasmid, which—unlike pLVPK—was conjugative, and one of these plasmids simultaneously harbored multiple AMR genes (*blaCTX-M-15*, *sul2*, *blaTEM-1*, *APH(3″)-Ib*, and *APH(6)-Id*). In the remaining three isolates lacking plasmid-borne *iro*, the transferred virulence module was integrated into the chromosome. In two of these three isolates, chromosomal integration also included *rmpA* and *peg-344*, while in one isolate only *peg-344* accompanied the *iro* cassette. To characterize the genetic context of these virulence loci, we analyzed their flanking regions. Structural analysis revealed that the mobilized *iro* cassette was bordered by IS1-family insertion sequences (IS1N and IS1D), indicating IS1-mediated recombination as the underlying mechanism.

Four isolates (4/96, 4.2%) lacked virulence plasmids– three were completely plasmid-free, and one retained a single AMR plasmid (IncC). In all four, virulence-associated loci were chromosomally integrated. Flanking regions analysis revealed that in two isolates obtained from the same patient (P2), the region containing the *iut* gene cluster was bordered by IS903, an IS5-family element, and was accompanied by perfectly matched 10-bp direct repeats located immediately upstream and downstream of the insertion, consistent with a classical insertion event. In the remaining two isolates, the virulence *iut* loci were also flanked by IS5-family elements; however, direct repeats were absent. Instead, each flanking boundary consisted of a conserved left-hand sequence shared across all genomes and a distinct but equally conserved right-hand sequence, suggesting recombination-driven integration rather than simple transposition.

A total of 70/96 (72.9%) isolates carried at least one plasmid associated with antimicrobial resistance, encompassing six major AMR plasmid types (Figure 5). Five of these 70 isolates possessed two distinct AMR plasmids simultaneously (IncFII/rep_cluster_1418; rep_cluster_1418/IncC; IncL/M; or IncX1/IncX3), indicating either multiple acquisition events or stable long-term coexistence supported by compatible replication systems. AMR plasmids varied in size and gene content, and belonged to IncFII, rep_cluster_1418, IncC, IncL/M, IncN, IncX1, or IncX3 incompatibility groups. Representative plasmid maps are shown in Figure 5. In 68 isolates, *sul2* was plasmid-borne, while in two isolates it was located exclusively on the chromosome. One isolate exhibited an atypical AMR gene distribution pattern, with extensive chromosomal integration of typically plasmid-associated genes (*catB3*, *dfrA14*, *qnrB*) and simultaneous *aac(3)-IIa*, *aac(6′)-Ib-cr*, *blaCTX-M-15*, and *blaOXA-1* presence on both plasmid and chromosome. Another isolate carried a fully assembled resistance plasmid that could not be assigned to any known incompatibility group; its replication locus belonged to rep_cluster_1418 (Figure 5), a family previously associated with multidrug-resistant *K. pneumoniae*.

Fusion plasmids combining virulence and resistance determinants were identified in 9/96 (9.4%) isolates (Figure 4). Seven of these plasmids shared a highly similar architecture combining IncFIB, IncFII, and IncHI1B replicons, consistent with recombination between a pLVPK-like virulence backbone and AMR plasmids. Two isolates carried an alternative fusion variant involving IncC together with IncFIB and IncHI1B. The presence of these virulence–resistance fusion plasmids highlights ongoing plasmid modularity and dynamic evolutionary processes within the K1/ST23 lineage.

### 3.3 Virulence determinants

All 96 UHB isolates belonged to the hypervirulent K1 capsular serotype and uniformly carried the O-antigen type O1⍺β, 2β, mirroring the global predominance of this O-type among K1 strains (92.3% in the global dataset). Virulence profiling showed an exceptionally homogeneous hypervirulent population: 95 isolates reached the maximal virulence score of 9, and a single isolate scored 8 due to the absence of *allS*. In comparison, the global collection exhibited broad variability, with virulence scores ranging from 1 to 9; however, highly virulent profiles predominated, as 2,019 isolates (82%) reached score 9.

Screening of nine hypervirulence-associated loci revealed an almost identical virulence gene repertoire within the UHB cohort. All isolates harbored *ybt* and *clb* organized within the chromosomal ICEKp10 integrative element, and *allS* was present in 95/96 isolates, consistently located within the allantoin utilization island. This pattern is concordant with the global dataset, where *ybt* positivity was also nearly universal (2,391/2,463), with ICEKp10 as the dominant variant (2,313 isolates), and *clb* was detected in 2,332 isolates, predominantly as CbST29. However, global strains exhibited markedly greater yersiniabactin and colibactin sequence types diversity (16 YbSTs and 16 CbSTs globally versus 8 and 2, respectively, in UHB). In UHB, YbST47 dominated (78%), whereas globally both YbST47 (*n* = 1,016) and YbST46 (*n* = 967) were common. The mobile element Tn7399 encoding the *mce* gene cassette was detected in all UHB isolates, again consistent with its high prevalence in the global dataset (93%). Similarly, *peg-344* was universally present in UHB isolates and was detected in 2,336 global isolates.

Plasmid-associated virulence determinants were widespread across the UHB collection: 83/96 isolates carried the canonical pLVPK-associated genes (*iuc*, *iro*, *peg-344*, *rmpA*, and *rmpA2*). Aerobactin (AbST1) and salmochelin (mostly SmST2) were present in all UHB isolates, with significantly less diversity than in the global dataset, where 12 AbSTs and 10 SmSTs were identified. Notably, AbST1 and SmST2 were also dominant globally (2,321 and 2,187 isolates, respectively), reaffirming the conserved nature of these loci in K1 hypervirulent clones.

Mucoid phenotype regulators, *rmpA* and *rmpA2*, were present in 96/96 (100%) UHB isolates. Although the UHB cohort displayed 12 different RmSTs, it remained less diverse than the global dataset (47 RmSTs identified globally). The globally dominant types, RmST26 and RmST40, were also prominent in the UHB dataset, indicating phylogenetic relatedness between local and globally circulating K1 hypervirulent lineages.

Collectively, comparison with the global K1/ST23 KP dataset confirms that UHB isolates form a highly conserved and homogenously hypervirulent population, with minimal virulence gene diversity and complete or near-complete representation of hallmark hypervirulence determinants. This contrasts with the broader global population, where greater heterogeneity exists despite similar overall virulence potential. Taken together, the UHB strains represent a tightly clustered subset of the globally dominant K1–O1⍺β, 2β hypervirulent lineage, characterized by maximal virulence scores, uniform pLVPK-associated loci presence, a consistent ICEKp10 background, and near-absolute prevalence of all key siderophore and mucoid-regulator genes.

### 3.4 Antimicrobial resistance profiles

Thirty distinct resistance gene profiles were identified across the 96 isolates. All isolates carried chromosomal *blaSHV-190* and the efflux transporters *oqxA* and *oqxB*. With a single exception, all isolates harbored chromosomal *fosA6*. The ESBL phenotype present in 62 isolates correlated with the presence of *blaCTX-M-15*, predominantly plasmid-borne, although in five isolates the gene was present on both plasmid and chromosome. No carbapenemase genes were detected, consistent with universal susceptibility to carbapenems. The phenotypic antimicrobial susceptibility testing results are provided in the Supplementary Table S3.

When compared with the global collection, which comprised 348 unique resistance profiles, the UHB isolates displayed substantially lower resistance gene diversity. Carbapenemase genes—detected in 374/2463 global isolates—were absent in the UHB cohort. In the global population, carbapenemases were dominated by Class A enzymes (140 isolates, including 130 *KPC-2*), followed by Class D (129 isolates, most frequently *OXA-48* in 104 isolates), whereas Class B metallo-β-lactamases were found in 4.3% of isolates (IMP 1.6%, NDM 2.7%). In contrast, core chromosomal determinants such as *blaSHV-190* (*n* = 2,430), *fosA6* (*n* = 2,461), and *oqxA/oqxB* (*n* = 2,453) were similarly widespread in both datasets. However, *blaCTX-M* genes were less common globally (blaCTX-M-15 was detected in 144/2463 global isolates, while any *blaCTX-M* variant was present in 294/2463 isolates) than in the UHB isolates, where *blaCTX-M-15* predominated among ESBL-producing strains.

## 4 Discussion

This study provides a comprehensive epidemiological and genomic characterization of hvKp isolates recovered at a tertiary-care hospital in the Czech Republic from the K1/ST23 lineage and places these findings within the global population context. From 570 screened isolates, 96 isolates originating from 82 patients were categorized into the K1/ST23lineage based on molecular and genotypic criteria, underscoring this lineage’s clinical and the need for systematic surveillance. It should be noted that isolates representation was not uniform across years. A substantial reduction in sampling occurred between 2020 and 2022—most markedly in 2020—due to COVID-19-driven prioritization of pandemic response. This constraint may have reduced detection sensitivity for hvKp during that period, an important consideration when interpreting temporal trends.

Our confirmation that the predominant hvKp subpopulation belongs to the K1/ST23 lineage aligns with global observations identifying ST23 as the most successful and widely disseminated hypervirulent clone (Russo and Marr, 2019; Zhu et al., 2021). This lineage is well known for causing severe community-acquired invasive infections, including pyogenic liver abscesses often complicated by metastatic spread such as endophthalmitis and meningitis, even in otherwise healthy individuals. K1 and K2 serotypes remain the most widespread capsular types linked to invasive disease (Kocsis, 2023; Mendes et al., 2023; Najafian et al., 2025). A hvKp hallmark is the combined expression of multiple virulence factors, including hypermucoviscosity, specific capsular serotypes, and accessory genetic markers such as *rmpA*, and siderophore genes (*iucABCD*). In our cohort, virulence profiling confirmed an exceptionally homogeneous hypervirulent population: 95 isolates achieved the maximal virulence score of 9, and a single isolate scored 8 due to the absence of *allS*. This high uniformity level closely parallels the global dataset, in which 96.6% of isolates exhibited a virulence score of 8 or 9, underscoring the worldwide predominance of highly virulent ST23/K1 KP clones. Similar patterns have been reported in Iran, where hvKp isolates associated with cryptogenic liver abscesses consistently belonged to ST23 or ST65 and carried a full virulence gene complement, including *rmpA*, *rmpA2*, *iucA*, and *iroB* (Sohrabi et al., 2022).

Core-genome phylogenetic analysis demonstrated that Czech isolates were dispersed across multiple branches of the global K1/ST23 KP phylogeny rather than forming a unified monophyletic cluster. This pattern suggests that the observed cases do not represent a local clonal expansion or outbreak but rather reflect repeated K1/ST23 KP strain being introduced into the Czech population over time. Comparable patterns have been reported in other European and Middle Eastern countries, indicating that hvKp dissemination is being driven by international travel, healthcare-associated transmission, and community carriage (Biedrzycka et al., 2022; Sohrabi et al., 2022; García-Cobos et al., 2025). The absence of tight clustering among UHB isolates underscores the importance of ongoing genomic surveillance to detect potential shifts toward clonal expansion or outbreak scenarios.

Historically, K1/ST23 KP isolates were considered highly susceptible to antibiotics. However, our results demonstrate notable resistance genes acquisition within the UHB isolates. Specifically, *blaCTX-M-15* predominated among ESBL-producing strains, eliminating third-generation cephalosporins as a treatment option. Chromosomal determinants such as *blaSHV-190*, *fosA6*, and *oqxA/oqxB* were universally present across the Czech and global K1/ST23 population. Although carbapenemase genes were absent in the UHB population, the presence of ESBL determinants and plasmid modularity raises concern for future hypervirulence and carbapenem resistance convergence, as documented in other regions (Shankar et al., 2020; Gálvez-Silva et al., 2024). Recent studies confirm that this convergence typically occurs through two evolutionary routes: hvKp strains acquiring resistance plasmids or classical MDR strains acquiring virulence plasmids (Mendes et al., 2023; Russo et al., 2024b; Tu et al., 2024). The emergence of K1/ST23 KP strains carrying both pLVPK-like virulence plasmids and carbapenemase genes such as blaKPC-2 has been reported in China, where these strains exhibited high mortality in animal models and triggered NF-κB–mediated hyperinflammatory responses (Tu et al., 2024). Similarly, Hernández et al. (2021) described an extensively drug-resistant K1/ST23 KP isolate in Spain co-producing *blaOXA-48*, *blaCTX-M-15*, and *armA*, carried on hybrid plasmids combining virulence and resistance determinants. These findings highlight the potential for similar evolutionary events in Europe (Cheong et al., 2016) and further demonstrate that even community-acquired K1/ST23 KP isolates can harbor plasmid-mediated *AmpC* β-lactamases alongside ESBL phenotypes, indicating that multidrug resistance in hvKp is not confined to hospital settings.

Plasmid analysis revealed substantial modularity and extensive genomic plasticity within the K1/ST23 KP plasmidome, consistent with the dynamic evolution previously described for *K. pneumoniae* (Han et al., 2025). Most isolates carried pLVPK-like IncFIB/IncHI1B virulence plasmids harboring *iucABCD*, *iroBCDN*, *rmpA*, *rmpA2* and *peg-344*, showing a conserved virulence backbone typical for hvKp (Chew et al., 2017; Hernández et al., 2021; Russo et al., 2024b; Jiang et al., 2025). Long-read sequencing proved essential to resolve these complex plasmid structures and to differentiate between plasmid-borne and chromosomally integrated virulence loci. Beyond the classical pLVPK architecture, our findings uncovered previously under-recognized modes of virulence gene mobility, highlighting virulence determinants fluidity in ST23. Most notably, we identified the *iro* gene cluster relocating onto a conjugative IncFII/rep_cluster_1418 plasmid, which in one isolate also carried multiple AMR genes. Co-localization of *iro*, *rmpA* and *peg-344* on a conjugative backbone constitutes a mechanistically important shift, providing a fully transmissible vehicle for major hypervirulence determinants normally restricted to the non-conjugative pLVPK plasmid. This architecture resembles emerging virulence–AMR hybrids reported in Asia and Europe (Chew et al., 2017; Hernández et al., 2021) and raises a realistic concern regarding horizontal hypervirulence dissemination into MDR classical *K. pneumoniae* backgrounds.

Three isolates further revealed the *iro* virulence module’s chromosomal integration mediated by IS1-family elements (IS1N/IS1D). These data refine earlier assumptions and demonstrate IS1-mediated recombination—rather than IS5-associated mechanisms—as the dominant pathway for *iro* mobilization. Because IS1 supports both plasmid–plasmid and plasmid–chromosome recombination, this mechanism substantially broadens the evolutionary routes through which virulence modules can be reorganized, stabilized or disseminated.

In contrast, *iut* (aerobactin) locus chromosomal integration was consistently associated with IS903 (IS5-family) elements, resulting in two mechanistically distinct outcomes. In two isolates, perfectly matched 10-bp direct repeats flanking the insertion were consistent with canonical IS5-mediated transposition (Nielsen et al., 2014). In two others, direct repeats were absent despite the presence of IS903, and integration occurred within conserved but non-identical flanking regions, indicating homologous recombination or plasmid–chromosome recombination rather than classical transposition (David et al., 2020; Onstead et al., 2024; Pan and Li, 2025). Together, these findings support the view that different virulence modules use distinct insertion-sequence families and distinct molecular mechanisms—IS1 drives *iro* mobility, while IS5 drives *iut* mobility.

Plasmid modularity extended beyond virulence loci. The cohort contained a structurally heterogeneous resistance plasmid set, including an untypeable rep_cluster_1418 plasmid, underscoring ST23 plasmids’ capacity to recruit novel AMR determinants. Nine virulence–resistance fusion plasmids were identified, seven combining IncFIB–IncFII–IncHI1B replicons and two representing IncC–IncFIB–IncHI1B variants. These mosaics are consistent with IS26- and IS-family–mediated recombination, which has been shown to merge non-conjugative virulence plasmids into conjugative AMR backbones (Tian et al., 2025). The presence of such hybrids in the Czech K1/ST23 KP population demonstrates that the lineage harbors the structural prerequisites for the emergence of MDR-hvKp, even in the absence of current carbapenemase acquisition.

Together, these findings illustrate that genomic plasticity in ST23 arises through the combined action of modular plasmid evolution, plasmid–plasmid fusion, and both recombinational and transpositional chromosomal integration events. This interplay between plasmids and the chromosome promotes the stabilization and dissemination of hypervirulence and antimicrobial resistance traits, amplifying the evolutionary potential of ST23 and complicating clinical and public-health responses.

Diagnostic challenges persist due to the historical reliance on hypermucoviscosity as an hvKp marker. Neumann et al. (2023) reported that only 0.69% of clinical isolates from a German tertiary-care center met molecular criteria for hvKp despite 7.6% being string-test positive, confirming that hypermucoviscosity alone is an unreliable marker. Their findings underscore the need for combined genotypic and phenotypic approaches, with aerobactin (*iutA*) emerging as the most stable marker of hypervirulence. Routine implementation of multiplex PCR targeting *rmpA*, *rmpA2*, *iutA*, and *magA*, alongside WGS for plasmid characterization, is recommended to improve detection accuracy.

Emerging evidence also suggests that capsule gene alterations may facilitate the convergence of hypervirulence and carbapenem resistance. Wang et al. (2022) demonstrated that insertion sequence-mediated disruption of the *wcaJ* gene in K1/ST23 KP isolates reduces capsule synthesis, compromising hypermucoviscosity but lowering fitness costs and enhancing conjugation efficiency for carbapenemase plasmids. This mechanism may accelerate CR-hvKp emergence by promoting plasmid acquisition while maintaining sufficient virulence for clinical infection.

The combination of extreme virulence and emerging antimicrobial resistance poses a major therapeutic and public-health challenge. Although all isolates in our cohort remained carbapenem-susceptible, the high ESBL production prevalence already narrows treatment options and increases reliance on last-line agents. In parallel, the convergence of virulence and transmissible genetic elements observed in this study highlights the urgent need for enhanced genomic surveillance, particularly focusing on the mobility of virulence modules that may accelerate MDR-hvKp emergence in Central Europe. Proactive infection control, together with rapid diagnostic tools capable of distinguishing hvKp from classical lineages, is essential for optimal clinical decision-making, early detection of metastatic complications, and timely containment of high-risk strains.

This study has limitations, particularly its single-center design, which might restrict generalizability. The absence of systematic national hvKp surveillance may lead to underestimating the true prevalence and diversity of hvKp lineages. Future work should include coordinated multicenter genomic surveillance to track transmission patterns, monitor evolutionary trajectories, and identify early convergence signs between hypervirulence and carbapenem resistance.

In conclusion, K1/ST23 KP strains circulating in the Czech Republic are genetically heterogeneous, highly virulent, and exhibit early signs of plasmid-mediated resistance acquisition. Although no carbapenemase genes were detected, the presence of ESBL determinants and fusion plasmids underscores the potential for convergence toward MDR-hvKp. Continuous genomic monitoring and targeted infection control measures are essential to prevent these high-risk pathogens emerging and disseminating.

## Data Availability

The datasets generated for this study can be found in the NCBI Sequence Read Archive (SRA) under accession numbers SRR36095863–SRR36095913 and SRR36095937–SRR36095981, within BioProject PRJNA675431.

## Footnotes

1
https://www.ncbi.nlm.nih.gov/

2
https://bigsdb.pasteur.fr/
