# Supplementary material for: Emergence and genomic characterization of hypervirulent ST23/K1 Klebsiella pneumoniae: local epidemiology and global context
Source: Front Microbiol. 2026 Feb 2;17:1758288. doi: 10.3389/fmicb.2026.1758288 (PMC12907322; doi:10.3389/fmicb.2026.1758288)
Supplement: Supplementary file 2 [file Data_Sheet_1.pdf]

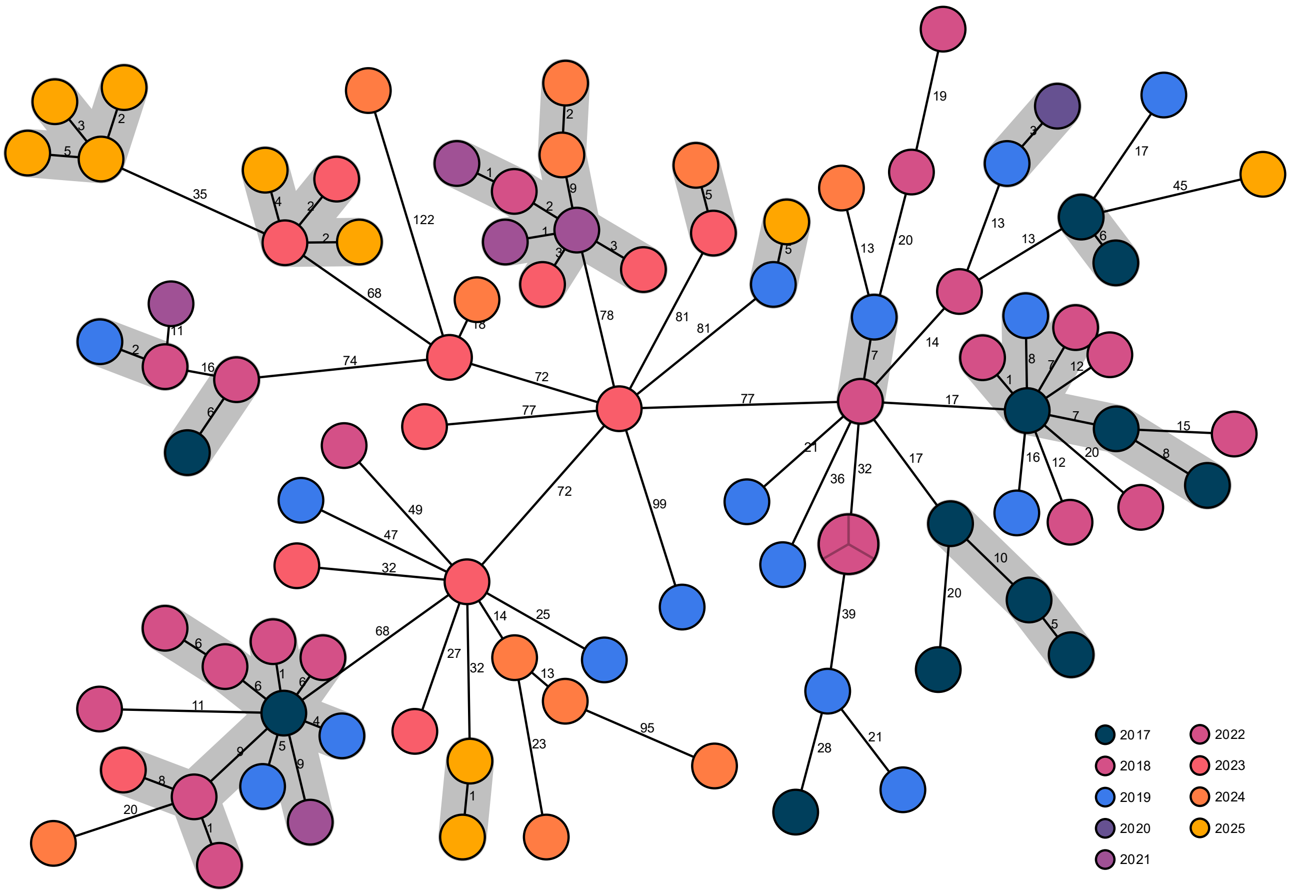

Figure S1. cgSNV minimum-spanning tree of 96 UHB K1/ST23 isolates. Node colors indicate collection year, and numbers shown on connecting edges represent SNV distances between linked isolates. Isolates belonging to the same phylogenetic cluster are shaded in grey.

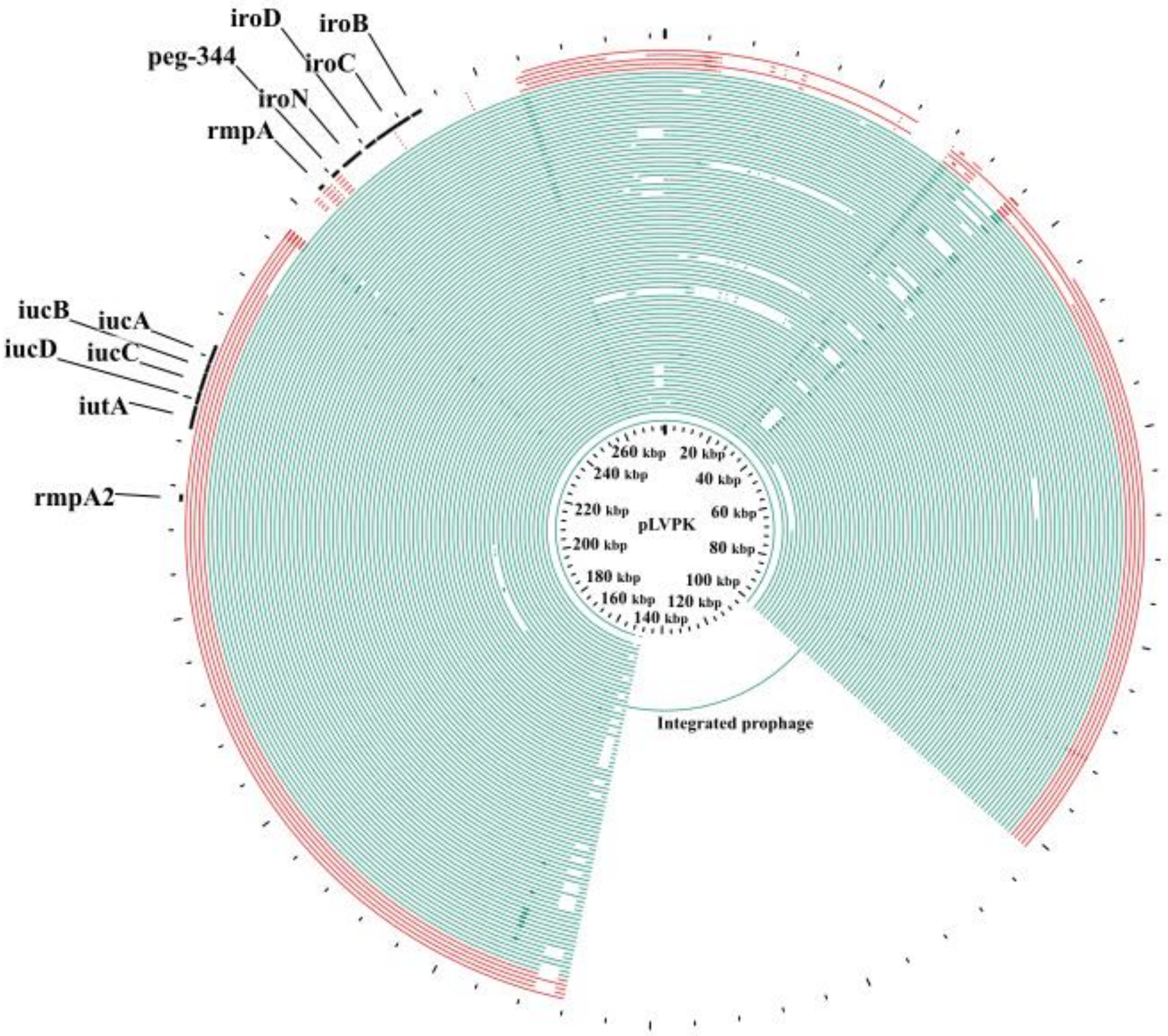

Figure S2. Proksee alignment of all 83 virulence plasmids detected in K1/ST23 UHB isolates. Annotated virulence genes are shown. Plasmids lacking the iro gene cluster are highlighted in red.
